# Territorial and Extraterritorial Trigeminocardiac Reflex: A Review for the Neurosurgeon and a Type IV Reflex Vignette

**DOI:** 10.7759/cureus.11646

**Published:** 2020-11-23

**Authors:** Daniel S Leon-Ariza, Juan S Leon-Ariza, Mayra A Gualdron, Jaime Bayona-Prieto, Fidias E Leon-Sarmiento

**Affiliations:** 1 School of Medicine, Santander University-UDES, Bucaramanga, COL; 2 Neuroscience, Mediciencias Research Group, Miami, USA; 3 Faculty of Medicine, Unicolsanitas, Bogota, COL; 4 Cirineo Research Group, Unicolciencias, Bucaramanga, COL; 5 Environmental Health, Florida International University, Miami, USA; 6 Neurology, Baptist Health South Florida, Miami Neuroscience Institute, Miami, USA; 7 Internal Medicine, National University, Bogota, COL

**Keywords:** trigeminocardiac reflex trigeminal nerve, spinal cord, neurophysiology, neuromonitoring, neuromodulation

## Abstract

The trigeminocardiac reflex (TCR) is a complex and, sometimes, fatal event triggered by overstimulation of the trigeminal nerve (TN) and its territorial and spinal cord branches. We reviewed and compiled for the neurosurgeon key aspects of the TCR that include a novel and straightforward classification, as well as morphophysiology, pathophysiology, neuromonitoring and neuromodulation features. Further, we present intraoperative data from a patient who developed extraterritorial, or type IV, TCR while undergoing a cervical surgery. TCR complexity, severity and unwanted outcomes indicate that this event should not be underestimated or overlooked in the surgical room. Timely TCR recognition in surgical settings is valuable for applying effective intraoperative management to prevent catastrophic outcomes.

## Introduction and background

The trigeminocardiac reflex (TCR) is a complex neurovascular reflex triggered by overstimulating the trigeminal nerve (TN) and its anastomosis. TCR often starts by awkwardly upregulating cardiovascular function followed by bradycardia and hypotension; if these aspects are not timely controlled hemodynamic dysfunction may escalate to cardiac arrest leading it, sometimes, to fatal outcomes. Although neurobiology of this reflex has been widely studied for centuries in animals, only recently it attracted scientific attention in human neurological surgery. As a consequence, human TCR neurobiology is still poorly understood, precluding it to identify and control this reflex in a timely manner during neurosurgical interventions [1,2].

Aiming to fill the gap of reflex assessment in the operating room, we recently devised a novel TCR classification to rapidly, easily, and accurately identify and assess the reflex in the surgical room. That classification was based on territorial and extraterritorial TN anatomical connections, and the patient's hemodynamic status (Table 1). Here, we review TCR variations and their alignment with current classification, reflex physiology as well as pathophysiology mechanisms supported by representative neurophysiological recordings. The framework provided here can help prevent wasteful workup, improve patient safety, and re-orient current practices.

**Table 1:** Trigeminocardiac reflex classification Class A is a TCR with HR alterations only, whereas class B is a TCR with MABP alterations only. eTN: extraterritorial trigeminal nerve, HR: heart rate, MABP: mean arterial blood pressure, TN: trigeminal nerve.

TCR Type	TN Branch	TCR Class	
I	Ophthalmic (V_1_)	A (HR)	B (MABP)
II	Maxillary (V_2_)		
III	Mandibular (V_3_)		
IV	Extraterritorial (eTN)		

## Review

Basic aspects

TCR is defined as dramatic bradycardia and marked hypotension commonly preceded by sudden and unexplained high blood pressure in the context of a normal or near-normal heart rate [1,2]. In a recent study, we found a reflex prevalence of 8.9% among 575 patients undergoing neurosurgical interventions. Heart rate and mean arterial blood pressure scores were independently altered during TCR elicitation. Of remark, these cardiovascular measures inversely correlated with each other with the clinically established reflex, which covaried with age [1,2]. TCR was triggered more often by surgical manipulation of the maxillary nerve; therefore, TCR type II was the most prevalent reflex type found when using the newly devised TCR classification. Importantly, this new classification method not only outlines the territorial TCR type according to the stimulated TN branch but also pinpoints the extraterritorial TN origin for the reflex such as that following spinal cord and limb stimulation [1,2].

Territorial TCR and variations

TCR is defined as dramatic bradycardia and marked hypotension commonly preceded by sudden and unexplained high blood pressure in the context of a normal or near-normal heart rate [1,2]. Territorial TCR was deﬁned according to TN anatomical branch distribution as type I, II, and III [1.2]. One, or more than one, of these TCR types may appear by stimulating the ophthalmic (Type I), the maxillary (Type II), and the mandibular (Type III) branch of the TN [1.2]. Hemodynamic alterations listed as TCR variations have also been reported following manipulations of craniofacial organs. Such variations include the oculocardiac reflex, the nasotrigeminocardiac reflex, and the maxillo-mandibulo cardiac reflex [3-6]. Integration of these variants into the new classification follows.

Oculo-cardiac reflex, reported in 1908 by Aschner and Dagnini, is triggered by stimulating the ophthalmic branch of the TN and anatomical areas surrounding the ocular globe [6,7]. This reflex corresponds to the TCR type I [1,2]. Nasotrigeminocardiac reflex, described by Kratschmer in 1870, occurs by stimulating the nasotrigeminal nerve, and mostly the nerve fibers located at the tip of the nose [8]. This reflex is also a TCR type I. Maxillo-mandibulo cardiac reflex, described by Loewinger and Shearer in 1987, is elicited by manipulation of maxillary and mandibular branches of the TN [9]. This reflex corresponds to TCR type II if the mandibular nerve, and TCR type III if the maxillary nerve is overstimulated [1,2].

Extraterritorial TCR

Extraterritorial TCR corresponds to the TCR type IV in the new classification [1,2]. Prevalence of this type of reflex in the operating room is unknown. Stimulation of lengthy neural connections located beyond TN territory would explain systemic hemodynamic changes reported during procedures performed at places far from the brainstem such as lower limbs and lumbosacral spinal cord manipulations [4,10,11]. Germane to this aspect, mean arterial blood pressure and vagal activity measured by spectral analysis of heart rate variability is modulated in response to thoracolumbar stimulation in cats [12], and to lumbar [13] and sacral stimulation in rats [14]. In humans, sacral afferent stimulation modulates efferent autonomic function via supraspinal integration of ventromedial medulla, hippocampus, insula, cingulate cortex, ventromedial prefrontal cortex, and cerebellum [15]. Hemodynamic modulation follows lumbar spinal cord manipulation in both injured and non-injured individuals [16,17]. These facts indicate that extraterritorial TCR induced by surgical procedures performed out of the craniofacial distribution of the three main branches of the TN can occur as shown below.

Macrophysiology

The physiology of this neurovascular event in humans undergoing neurosurgical procedures is still in development; data collected from several scenarios provide some enlightenment summarized as follows: First, overstimulation of mechanical receptor-associated trigeminal afferents at the outer layers of the cranium, the epicranium and meninges during pterional and frontotemporal craniectomy may trigger the reflex [18,19]. Second, transsphenoidal, retrosigmoid and subtemporal surgical approaches may also induce the TCR due to manipulations of TN and its anastomosis from the columella and zygoma [3,20]. Third, due to the high TN density, transsphenoidal, presigmoid and middle fossa approaches may also trigger TCR following removal of pituitary adenomas, petroclival masses, and meningiomas located in middle fossa [21,22]. Fourth, clipped aneurysms, carotid arteries exploration, and exposure of the cavernous sinus may trigger TCR due vasomotor dysregulation [23-26]. TCR has also been reported during the removal of nasopharyngeal angiofibromas, and in patients undergoing percutaneous embolization used for correcting dural arteriovenous fistulas [24,27]. Fifth, percutaneous needles placed near the foramen ovale, the place where the mandibular branch exits the skull, may also induce TCR [24,26]. Sixth, post-craniectomy implanted vacuum systems used to facilitate extradural-subgaleal drains have induced episodes of bradycardia, hypotension, and intracranial hypotension.32 Seventh, awake craniotomies may induce hemodynamic alterations that could lead to TCR [28]. Eight, spine surgical procedures up regulates neural transmission of lengthy TN anastomosis, and can trigger extraterritorial TCR by overshooting TN homeostasis at the brainstem [4].

Microphysiology

A central nanogenerator involved with TCR elicitation is believed to exist at the pons and the medulla; this generator senses afferent neural stimulation upon nerve fiber myelination status [29]. For instance, myelinated A-beta fibers are numerous in the supraorbital nerve innervating the forehead; however, they are sparse in the tip of the nose and are absent in the cornea [30,31]. In contrast, unmyelinated A-delta and C fibers are abundant in the nasotrigeminal nerve innervating the tip of the nose, and in the long ciliary nerve innervating the cornea [32]. This nerve fiber distribution within the ophthalmic branch is also found in the maxillary and mandibular branches of the TN [29]. Thus, axon diameter and associated nerve myelination explains, in part, that afferent neural stimulation applied to a particular nerve branch would down modulate differently the aforementioned brainstem generator.

**Figure 1:**
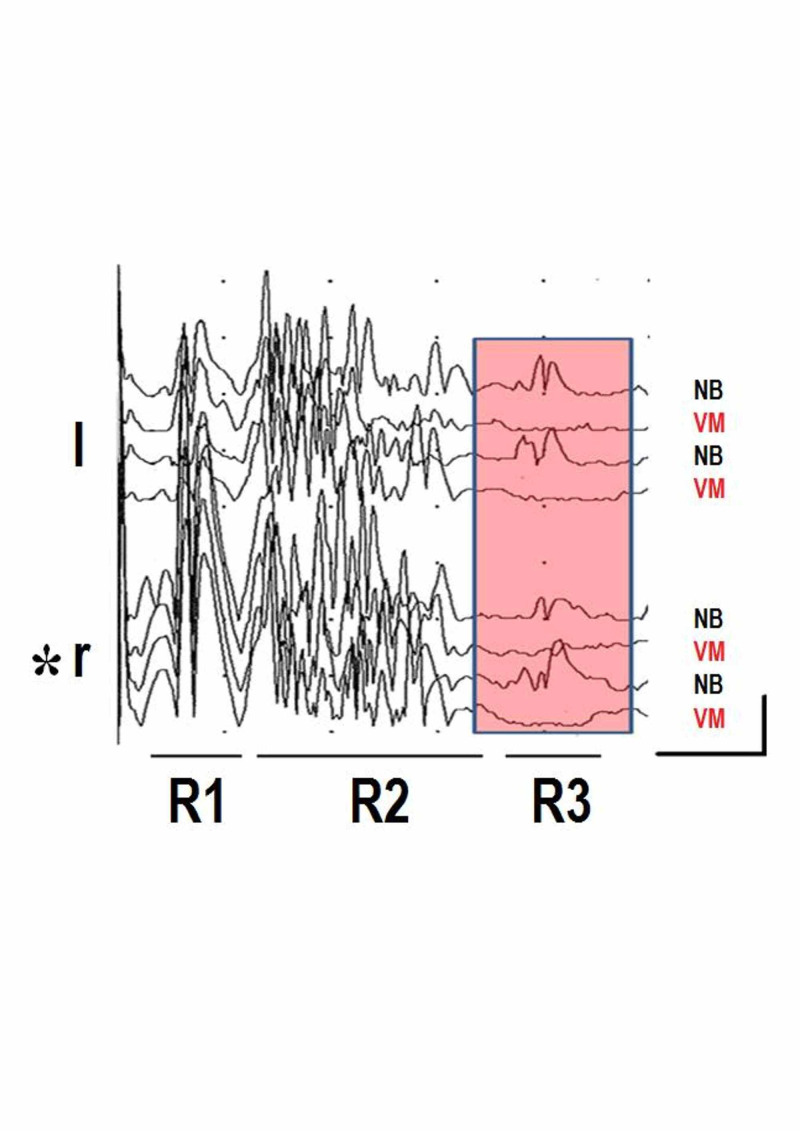
Blink reflexes and valsalva maneuver Four traces recorded from left (l) and Right (r) orbiculari oculi muscles after applying electrical stimulation of 0.2 ms duration at six times sensory threshold at the supraorbital nerve (asterisk).  Nociceptive A beta (R1), nociceptive A delta (R2), and ultra-nociceptive C fibers (R3) related responses were induced in a 36-year-old healthy male. Shaded area shows R3 response modulation during normal NB and while doing VM. Horizontal: 200 ms, vertical: 20 uV. NB: normal breathing, VM: Valsalva maneuver.

Detailed physiological studies demonstrated that myelinated primary afferents from animals with frontal eyes reach lamina V of the rostral part of the subnuclei caudalis of the spinal trigeminal nucleus, and lamina III/IV of the spinal dorsal horns [33]. Unmyelinated A-delta fibers, in turn, predominate in lamina I of the spinal dorsal horn, with few terminals existing in lamina IIa [34]. Single-unit microstimulation of neural afferents along with psychophysical studies unveiled human nociceptors activity arising from lower and upper limbs [35]. This nerve stimulation reaches supraspinal structures via spinal cord with conduction velocities similar to touch afferents [35]. Unmyelinated C nociceptive fibers project primarily to the ipsilateral superficial laminae I and II of the ventral medullary dorsal horn at the level of the area postrema, paratrigeminal nucleus, nucleus tractus solitarius and the Botzinger complex [36]. Noteworthy, this brainstem neural complex integrates both autonomic function and afferent sensory TN stimulation in humans (Figure 1) [37].

**Figure 2:**
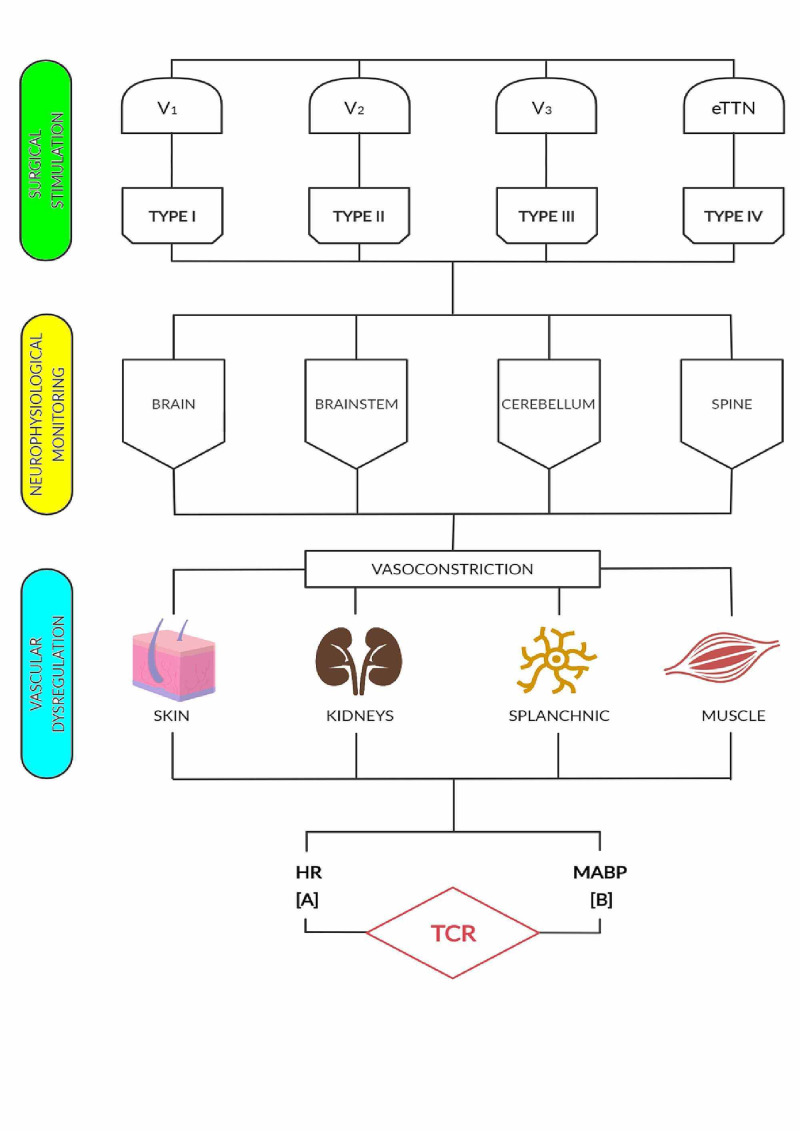
Schematic diagram depicting of hemodynamic neuromodulation of TCR elicited by territorial and extraterritorial TN stimulation BP: blood pressure; CN: cranial nerve; eTTN: extraterritorial trigeminal nerve, HR: heart rate; TCR: trigemino cardiac reflex; TN: trigeminal nerve. V1: ophthalmic branch, V2: maxillary branch, V3: mandibular branch. For types I, II, III and IV, and classes A and B definitions see text.

Territorial and extraterritorial TN afferent stimulation activates interneuronal connectivity at the nucleus of tractus solitaries and induces rostro-ventro-lateral excitation of the medulla. In this latter place, rich neurons of oxygen-sensitive sympathetic neurons exist [38,39]. These neurons and their associated reticular formation interneurons activate brainstem neural connections involved with sensory gating that filter incoming stimulus [39,40]. When these interneurons and associated pathways cannot compensate neural overshooting originated by aberrant afferent stimulation, they down regulate efferent neuronal and interneuronal firing. As a result, negative cardiac inotropism leads to bradycardia, hypotension, and in some cases, asystole. It has to be highlighted that cardiovascular responses following territorial and extraterritorial TN stimulation differ from classical vasovagal syncope. Indeed, during TCR elicitation, deregulation of inflammatory biomarkers such as IL-2, IL-17A and TNFα, and erratic behavior of metabolites such as norepinephrine, pancreatic polypeptide and acetylcholine happens in a very complex and not fully understood interplay [14]. Simultaneously, vasoconstriction first occurs in the skin, muscle, splanchnic vascular bed and kidneys, and diastolic and systolic blood pressures increase. As a result, cardiac contractility reduction happens (Figure 2) [41]. Vasovagal syncope, on the other hand, starts with prominent features of bradycardia and vasodilatation, rather than vasoconstriction or high blood pressure [41].

Neuromonitoring

We searched in PubMed and Scielo databases on surgical neurophysiological reports and TCR in humans returning no entries. Here, we present a 54-year-old male patient, who underwent an elective posterior C3-C7 laminoplasty after signing the consent form. This patient developed an extraterritorial TCR during the surgical procedure. To our knowledge, this is the first case reported in the literature from a human patient presenting a type IV TCR.

**Figure 3:**
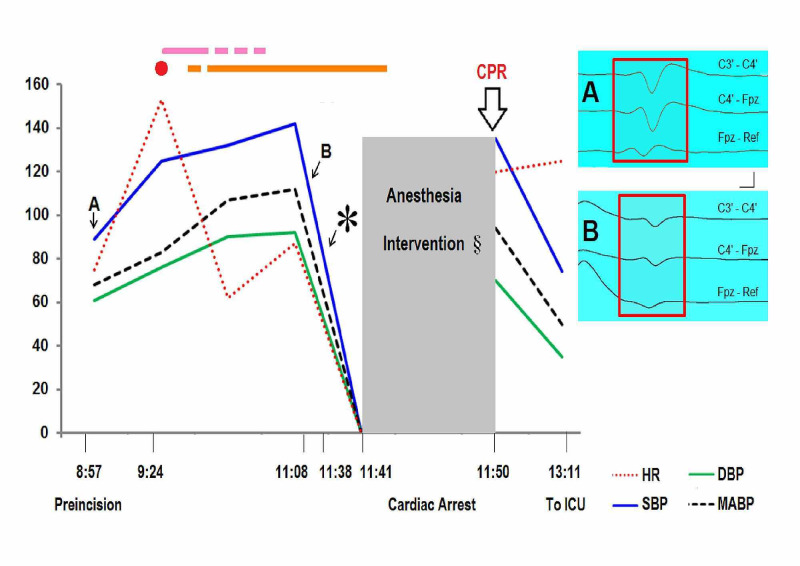
Extraterritorial TCR and somatosensory evoked potentials SSEPs were obtained by stimulating A beta fibers of the left ulnar nerve 200 times at 50 mA. Representative N20 recordings (A) were within normal limits at pre-incision time (8:57). There was a 50% reduction of N20 amplitude decrement and more than 10% of N20 latency prolongation in both cortical and subcortical recordings (B) during laminoplasty (11:08). Surgical neurophysiologist noticed the neurosurgeon, who continued with the surgical procedure despite the advice. Three minutes after SSEP became undetectable (11:38) cardiac arrest happened. Cardiopulmonary resuscitation procedures were applied (11:50) (thick arrow). Patient successfully recovered and transferred to the ICU. X-axis: military time not at actual scale. Y-axis: numeric values represent DBP, SBP, and MABP in mm Hg, and HR in beats/min. Red dot: incision, pink line: exposure, orange line: laminoplasty. DBP: diastolic blood pressure, HR: heart rate, ICU: intensive care unit, MABP: mean arterial blood pressure, SBP: systolic blood pressure, *: patient develops cardiac arrest, §: Lowering anesthesia medications (remifentanil and propofol) was unsuccessful. Vertical: one microvolt; horizontal: five milliseconds.

Using standard techniques, transcranial motor evoked potentials and somatosensory evoked potentials (SSEPs) were elicited (Elite®, Cadwell, Kennewick, WA, USA) [42,43]. In brief, the brain motor cortex was bilaterally stimulated using corkscrew electrodes placed approximately 1-2 cm anterior and medial to C3 and C4 scalp positions for transcranial stimulation. Motor evoked potentials were bilaterally recorded at the deltoid, biceps, triceps, abductor digit minimi, tibialis anterioris, and abductor halluxis muscles using subdermal paired needle electrodes. Motor evoked responses obtained pre-incision remained stable during the surgical procedure (data not shown). C3’-C4’, C4’-Fpz and Fpz-reference derivations were the recording montage selected to record the median nerve cortical SEPs. Representative data showing down regulation of SSEPs noticed during the TCR are presented in Figure 3.

These findings, and the absence of intraoperative cerebrovascular issues, ruled out a neurogenic shock as well as potential damage to corticospinal pathways during the surgery. These latter findings are aligned with the fact that long tracts such as those composing the corticospinal tracts are relatively resistant to reduced blood flow [44]. Pertinent to our findings, posterior tibial nerve SSEP disappears during controlled hypotension induced before spinal instrumentation [45]. Importantly, SSEP waveforms typically return after reduction of traction, and following mean arterial blood pressure increase [45]. Thus, SSEPs alteration found in our vignette may be an early biomarker for detection of, at least, extraterritorial TCR induced by manipulations of neural pathways anatomically located far from the brainstem.

Neuromodulation

The risk-benefit ratio to prevent TCR elicitation should be discussed and weighted while planning neurosurgical interventions. Anesthesia blockage of the scalp nerves and dura mater can prevent TCR [46]. The endoscopic-endonasal transsphenoidal approach used for resection of pituitary tumors have demonstrated to decrease hemodynamic alterations during patient recovery, and as consequence possibilities of triggering TCR [47]. Removal of abscesses or empyema drainage should be carefully done since strenuous neural activity may induce the reflex [27]. Attention should also be given to low neural thresholds existing in patients with epilepsy since bradycardia is often noticed while doing combined temporal lobectomy with amygdalo-hippocampectomy [48]. Hypoxia, hypercarbia, and acidosis should be carefully monitored since acid-base alterations may trigger TCR [49]. Spinal cord anesthesia can also trigger extraterritorial TCR, or type IV reflex [8]. Careful manipulation of the columellar area is also recommended during nasal surgical procedures since it can induce TCR [20]. Clinically established reflex can often be refractory to atropine used to modulate bradycardia; hence, alternative medications (e.g., adrenaline) should be considered to restore cardiovascular function [14,50]. Overall, cessation of neural stimulation is the best advice for preventing unwanted outcomes induced by the reflex. Should TCR become unstoppable, early implementation of advanced care life support is crucial to avoid further complications, as shown in the aforementioned vignette. 

## Conclusions

The rather low frequency that TCR is cared for in standard operating room checklist symptoms may lead to downplaying timely intraoperative management of the reflex. TCR can be a critical and devastating event that should be properly classified, timely approached, and wisely corrected. Surgical neurophysiological monitoring could be a useful tool to timely predict reflex elicitation. TCR complexity and severity indicates that this event should never be underestimated or overlooked, otherwise fatal outcomes may appear.
